# Resveratrol acts via the mitogen-activated protein kinase (MAPK) pathway to protect retinal ganglion cells from apoptosis induced by hydrogen peroxide

**DOI:** 10.1080/21655979.2021.1954742

**Published:** 2021-08-10

**Authors:** Ming-jing Ye, Ni Meng

**Affiliations:** Department of Pharmacy, Xiangyang Central Hospital, Affiliated Hospital of Hubei University of Arts and Science, Xiangyang, Hubei, China

**Keywords:** Resveratrol, retinal ganglion cells, apoptosis, oxidative stress, mapk pathway

## Abstract

The current study investigated the ability of resveratrol to protect RGC-5 retinal ganglion cells in culture against H_2_O_2_-induced apoptosis and the underlying mechanism of protection. RGC-5 cells were pre-exposed to resveratrol (5, 10, or 20 μM), followed by 200 μM H_2_O_2_. Cell viability and apoptosis were detected to assess the cell growth, and expression levels of apoptosis-related and MAPK cascade-associated proteins were determined using western blotting. Levels of reactive oxygen species and mitochondrial membrane potential were also tested, as well as the activities of superoxide dismutase (SOD), catalase (CAT), and glutathione S-transferase (GSH). At a concentration-dependent way, resveratrol reversed H_2_O_2_-induced increases in expressions of cleaved caspase-3 and cleaved caspase-9, production of ROS, loss of mitochondrial membrane potential and the expressions of p-p38, p-ERK, and p-JNK. It also promoted the activities of SOD, CAT, and GSH. Furthermore, the agonists of p38, ERK, and JNK partially weakened the protective effects of resveratrol against H_2_O_2_-induced apoptosis in RGC-5 cells. Thus, resveratrol can protect retinal ganglion cells against H_2_O_2_-induced apoptosis by suppressing MAPK cascades. The drug therefore shows potential for preventing glaucoma.

## Graphical abstract

**Figure:**
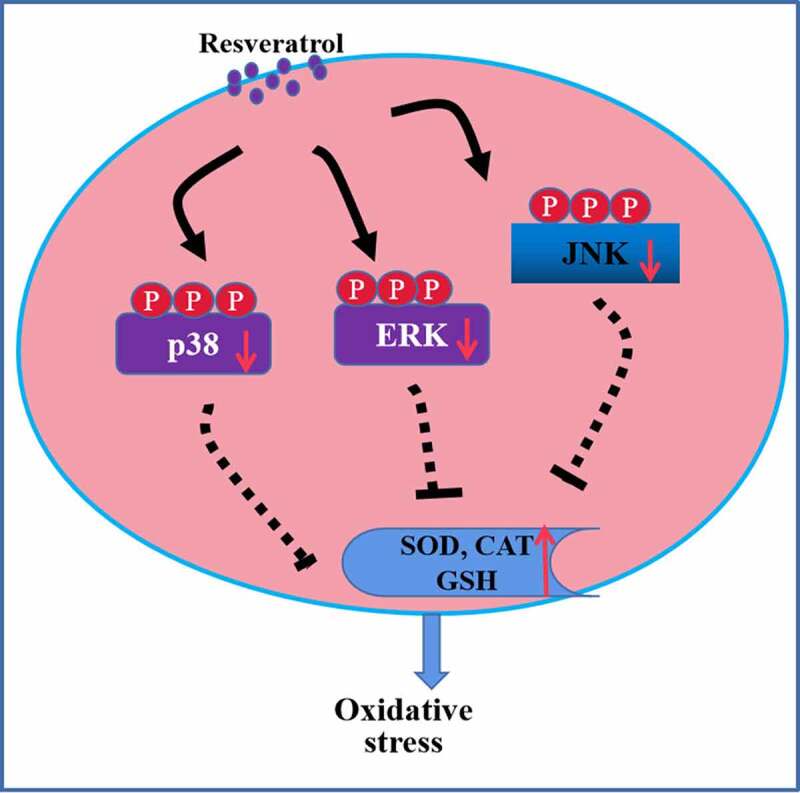

## Introduction

Glaucoma refers to a class of diseases characterized by progressive degeneration involving the apoptosis of retinal ganglion (RGG) cells [1]. Glaucoma was found to lead to blindness, its prevalence is rising, and it is estimated to cause optic neuropathy in 60 million individuals worldwide [2]. Oxidative stress, which is caused by malfunction of the intracellular antioxidant system, could lead to the abnormal production of reactive oxygen species (ROS) and overload antioxidant capacity [3]. It is well known that oxidative stress is an important factor to induce cell apoptosis, promote ROS production and suppress antioxidant enzyme activity, thus resulting in cell injury [4]. ROS-induced oxidative status and apoptosis are considered key drivers of glaucoma [5,6]. The main intracellular ROS is hydrogen peroxide (H_2_O_2_), which can activate many signaling pathways, including those involving apoptosis-associated signal, mitogen-activated protein kinases (MAPKs), and inflammation-related cascades; in this way, it promotes RGG cell death and ultimately glaucoma [7–9]. Thus, protecting RGG cells against ROS-stimulated apoptosis may effectively mitigate or even prevent glaucoma.

Resveratrol is a natural phytoalexin widely distributed in cereals, fruits, and vegetables [10] and it is a major component of red wine. Resveratrol exerts anti-cardiovascular, phytoestrogenic and anti-oxidant effects [11,12]. It can delay the onset and progression of glaucoma as well as other age-related ocular diseases (cataracts, diabetic retinopathy, macular degeneration) by inhibiting oxidative stress-induced cell apoptosis [13]. Resveratrol can also prevent retinal ganglion cell loss and attenuate gliosis-related inflammation resulting from ischemia-reperfusion injury [14]. How resveratrol prevents oxidative stress remains poorly understood.

The present study further explored the roles of resveratrol in H_2_O_2_-induced oxidative stress in RGC-5 retinal ganglion cells in culture and the mechanisms underlying the anti-apoptotic effects.

## Material and methods

### Reagents

Resveratrol, PD169316, FR180204, SP600125, anisomycin, curcumin and the fluorescent dye H2DCF-DA were provided by Gibco (Grand Island, NY, USA). Dimethyl sulfoxide (DMSO), H_2_O_2_, and the CCK8 kit were provided by Gibco (Grand Island, NY, USA). Antibodies against cleaved caspase-3, cleaved caspase-9, phospho-p38 (p-p38), phospho-ERK (p-ERK), or phospho-JNK (p-JNK) were provided by Abcam (Grand Island, USA). Annexin V/FITC kit was provided by eBioscience (Bender MedSystems, Vienna, Austria). Kits to assay catalytic activity of superoxide dismutase (SOD), catalase (CAT), and glutathione S-transferase (GSH) were obtained by Abcam (Grand Island, USA).

### Cell culture

RGC-5 cells were grown in DMEM/F12 medium which includes 10% fetal bovine serum at 37°C in an atmosphere of 5% CO_2_ in saturated humidity. Cultures in logarithmic growth phase were utilized for subsequent experiments. Cells were routinely subcultured every 2–3 days.

### CCK8 assay

After RGC-5 cells were exposed to H_2_O_2_ and resveratrol, the culture medium was removed completely. Then 100 μL of medium containing CCK8 reagent (10 μL) was added into each well for a 2-h incubation at 37°C under saturated humidity with 5% CO_2_. The optical density (OD) at 540 nm was measured using a microplate reader (Bio-Rad, Hercules, CA, USA). Relative cell viability (%) was defined as OD_experiment/_OD_control_ × 100%.

### Cell apoptosis

Proportions of apoptotic RGC-5 cells were determined by staining. In brief, cells were cultured in 6-well plates for 12 h. then were preincubated with the indicated doses of resveratrol for 4 h, exposed to 200 μM H_2_O_2_, then collected and rinsed twice with ice-cold PBS. RGC-5 cells were resuspended in 1× binding buffer, then 5 μL FITC-labeled annexin V and 10 μL propidium iodide (PI) were added and the mixture was incubated for 15 min at room temperature in the dark. The stained cells were analyzed using flow cytometry (Becton Dickinson, San Jose, CA, USA).

### ROS production

Intracellular ROS generation was measured by the ROS-specific fluorescent dye H2DCF-DA. RGC-5 cells were pre-exposed to the indicated doses of resveratrol for 4 h, exposed to 200 μM H_2_O_2_, rinsed twice with PBS, incubated with H2DCF-DA for 30 min under dark environment. Then cells were recovered and analyzed using flow cytometry.

## *Mitochondrial membrane potential (*Δψm*) and enzyme activity*

Effects of resveratrol on Δψm in H_2_O_2_-exposed cells were analyzed using rhodamine 123 as reported [15]. Activity of SOD, CAT, and GSH was evaluated using a colorimetric kit as reported [15].

### Levels of apoptotic and signaling proteins

After exposure to H_2_O_2_ and resveratrol, total protein in RGC-5 cells was harvested by splitting cells. Then BCA protein assay kit (Sangon Biotech Co., Ltd. Shanghai, China) was utilized to test the content of protein in each sample. Proteins were separated completely and transferred, then strips were incubated by 5% skim milk to block nonspecific binding sites. Subsequently, bands were incubated overnight with the primary antibodies (all diluted 1:1000) against the following proteins: cleaved caspase-3 (Abcam, #ab2209), cleaved caspase-9 (Abcam, #ab1160), p-p38 (Abcam, #ab1094), p-ERK (Abcam, #ab3240), or p-JNK (Abcam, #ab3210). After incubation, strips were rinsed by PBS-Tween 20, then the second antibody (diluted 1:1000) was used to incubate these blots. Protein expressions in strips were exposed by a exposure system (BioRad, California, USA).

### Statistical analysis

Results are represented as average ± SD. The differences among two groups were compared by Welch’s *t* test, and multi-group comparison was performed using ANOVA. Differences associated with *P*< 0.05 were regarded as significant. Statistical analysis was performed using GraphPad statistical software 6.0 (GraphPad Prism, Chicago, IL).

## Results

In our study, we supposed that resveratrol could improve the oxidative injury in RGC-5 cells induced by H_2_O_2_ by inactivating MAPK pathways. To confirm the protective effects of resveratrol against oxidative damage in H_2_O_2_-treated cells, we explore its effects on cell proliferation, apoptosis, and oxidative stress in H_2_O_2_-exposed cells. Next, the potential roles of MAPK pathways in RGC-5 cells were observed.

### Resveratrol increases the viability in H_2_O_2_-treated RGC-5 cells

**Figure 1.**
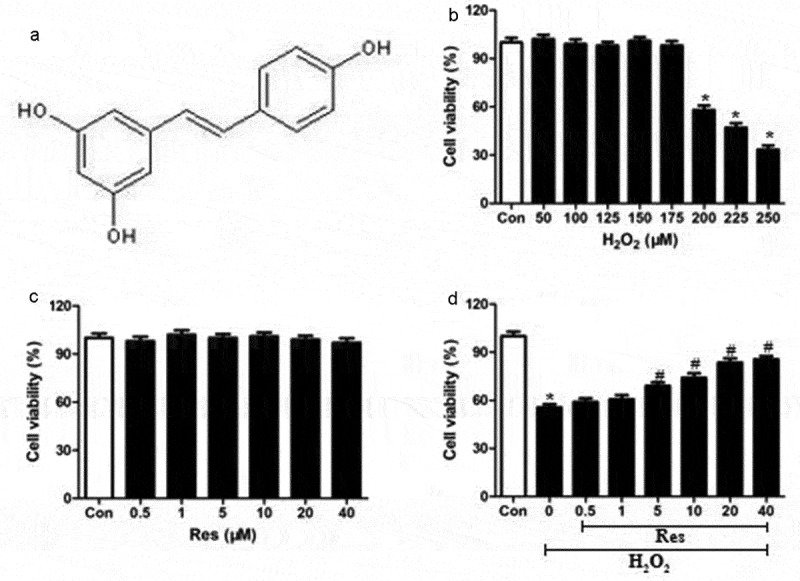
Ability of resveratrol to reduce H_2_O_2_-induced killing of RGC-5 cells. (a) structure of resveratrol (res). (b) RGC-5 cells were exposed for 24 h to gradient doses of H_2_O_2_ (50–250 μM). (c) RGC-5 cells were treated for 24 h with resveratrol at 0.5–40 μM. (d) RGC-5 cells were pretreated for 4 h with resveratrol at 0.5–40 μM, then 200 μM H_2_O_2_ for 24 h. Cell viability was assessed using the CCK8 assay. *P< 0.05 vs control group, ^#^*P*< 0.05 vs H_2_O_2_-treated group

First, we established that our cell culture system was functioning. The molecular structure of resveratrol was shown in Figure 1a. We showed that exposure to >200 μM H_2_O_2_ dramatically decreased viability of RGC-5 cells (Figure 1b), so 200 μM H_2_O_2_ was was utilized for the subsequent experiments. We also showed that incubating RGC-5 cells for 24 h in resveratrol concentrations from 0.5–40 μM did not cause significant cytotoxicity (Figure 1c).

### Resveratrol promotes the growth of H_2_O_2_-exposed RGC-5 cells

Preincubating RGC-5 cells for 4 h with resveratrol at 0.5–40 μM and then exposing them to H_2_O_2_ significantly increased viability at a concentration-dependent way (Figure 1d). These findings indicate that resveratrol can efficiently prevent the H_2_O_2_-induced death of RGG cells.

### Resveratrol prevents H_2_O_2_-induced apoptosis of RGC-5 cells

**Figure 2.**
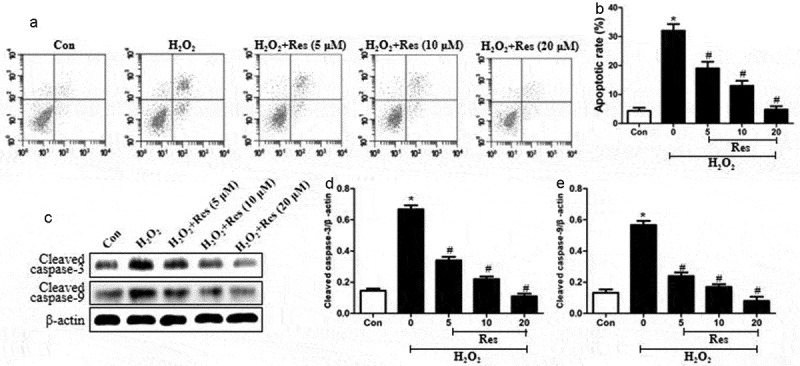
Ability of resveratrol to inhibit H_2_O_2_-induced apoptosis of RGC-5 cells. Cells were pretreated for 4 h with resveratrol (Res) at 5, 10, or 20 μM, then exposed to 200 μM H_2_O_2_ for 24 h. (a-b) Cell apoptosis was assessed using flow cytometry. (c-e) Levels of cleaved caspase-3 and cleaved caspase-9 were detected using western blots. *P< 0.05 vs control group, ^#^*P*< 0.05 vs H_2_O_2_-treated group

Exposing RGC-5 cells to 200 μM H_2_O_2_ substantially increased the proportion of apoptotic cells, which resveratrol reversed at concentration-dependent way (Figure 2a-b). Exposing cells to H_2_O_2_ also stimulated expressions of cleaved caspase-3 and cleaved caspase-9, which resveratrol reversed (Figure 2c-e). These data suggest that resveratrol can protect RGG cells against H_2_O_2_-stimulated apoptosis.

### Resveratrol reverses H_2_O_2_-stimulated intracellular generation of ROS and loss of Δψm in RGC-5 cells

**Figure 3.**
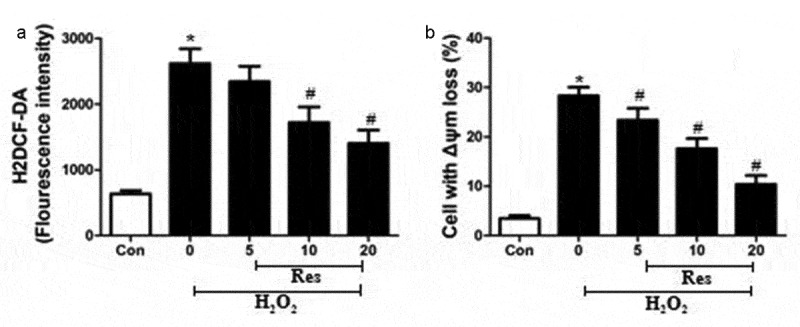
Ability of resveratrol to inhibit H_2_O_2_-stimulated generation of ROS and loss of Δψm in RGC-5 cells. Cells were pre-exposed for 4 h to resveratrol (Res) at 5, 10, or 20 μM, then exposed to 200 μM H_2_O_2_ for 24 h. (a) Cells were stained with H2DCF-DA at 37°C for 30 min in the dark, then analyzed using flow cytometry. (b) Cells were treated with rhodamine 123 for 30 min at 37°C, then analyzed using flow cytometry. *P< 0.05 vs control group, ^#^*P*< 0.05 vs H_2_O_2_-treated group

Exposing RGC-5 cells to 200 μM H_2_O_2_ significantly increased intracellular production of ROS, which resveratrol reversed in at a concentration-dependent way (Figure 3a). The H_2_O_2_ also dramatically increased the percentage of cells showing loss of Δψm, a marker of early apoptosis [14], which resveratrol reversed at a concentration-dependent way (Figure 3b).

### Resveratrol increases the activities of antioxidant enzymes in H_2_O_2_-incubated RGC-5 cells

**Figure 4.**
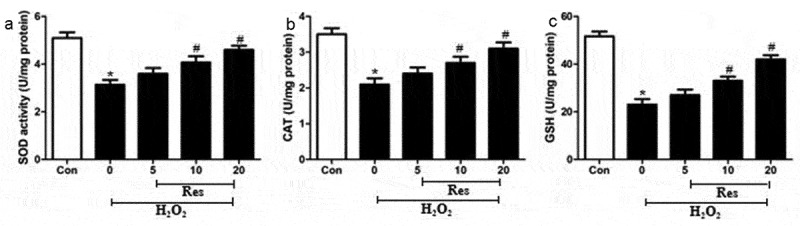
Ability of resveratrol (Res) to enhance the activities of SOD, CAT, and GSH in RGC-5 cells exposed to H_2_O_2_. Cells were pre-exposed for 4 h to resveratrol at 5, 10, or 20 μM, then exposed to 200 μM H_2_O_2_ for 24 h. The activities of SOD (a), CAT (b), and GSH (c) were assayed. *P< 0.05 vs control group, ^#^*P*< 0.05 vs H_2_O_2_-treated group

We examined whether resveratrol may exert its protective effects in part by activating radical-scavenging enzymes in retinal ganglion cells, primarily SOD, CAT, and GSH. Indeed, exposing RGC-5 cells to 200 μM H_2_O_2_ significantly decreased the activity of all three enzymes, which resveratrol reversed at a dose-dependent manner (Figure 4a-c).

### Resveratrol suppresses MAPK pathways in H_2_O_2_-treated RGC-5 cells

**Figure 5.**
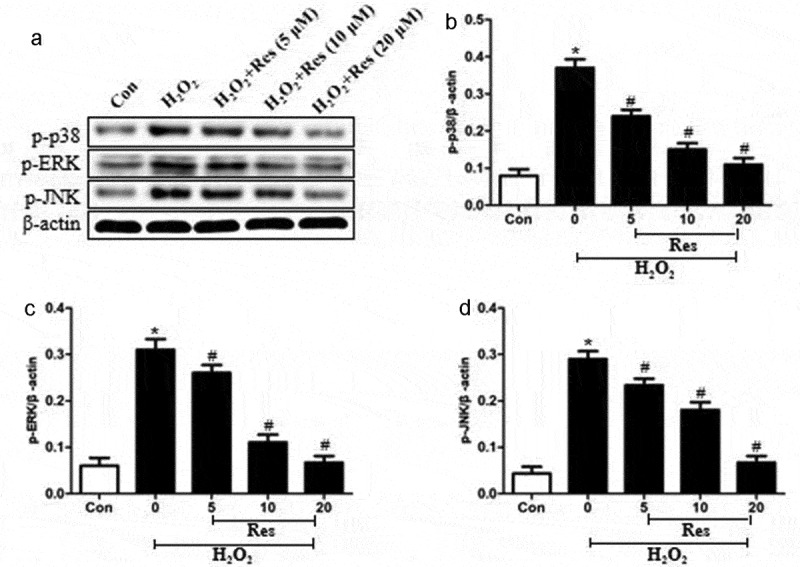
Ability of resveratrol to reduce levels of p-p38, p-ERK, and p-JNK in H_2_O_2_-treated RGC-5 cells. Cells were pre-exposed for 4 h to resveratrol (Res) at 5, 10, or 20 μM, then exposed to 200 μM H_2_O_2_ for 24 h. Levels of p-p38, p-ERK, and p-JNK were estimated by western blot. *P< 0.05 vs control group, ^#^*P*< 0.05 vs H_2_O_2_-treated group

Exposing RGC-5 cells to 200 μM H_2_O_2_ significantly increased levels of p-p38, p-ERK, and p-JNK, which resveratrol reversed at a dose-dependent way (Figure 5a-d). Our findings suggest that resveratrol suppresses H_2_O_2_-induced apoptosis of RGC-5 cells by inhibiting MAPK cascades.

**Figure 6.**
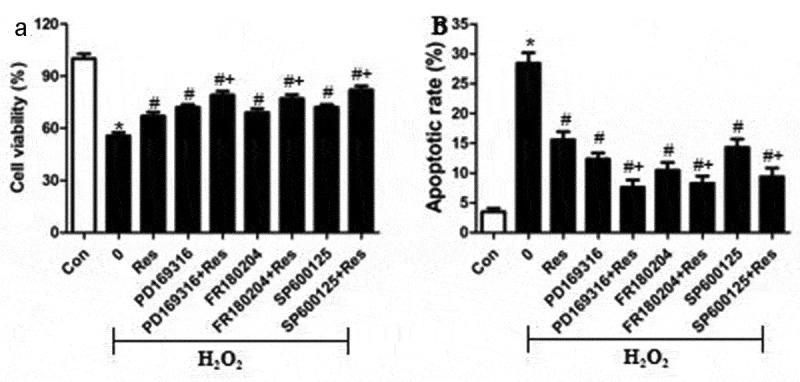
MAPK signaling inhibitors mimic and reinforce the effects of resveratrol on H_2_O_2_-induced apoptosis of RGC-5 cells. Cells were pre-exposed for 4 h to resveratrol (Res, 10 μM), PD169316 (1.25 μM), FR180204 (1.25 μM), or SP600125 (1.25 μM), or the combination of resveratrol with each of these inhibitors. Then cells were treated for 24 h with 200 μM H_2_O_2_. (a) Cell viability was detected using the CCK8 assay (b) Apoptosis was analyzed using flow cytometry. *P< 0.05 vs control group, ^#^*P*< 0.05 vs H_2_O_2_-treated group, ^+^*P*< 0.05 vs H_2_O_2_+ Res group

**Figure 7.**
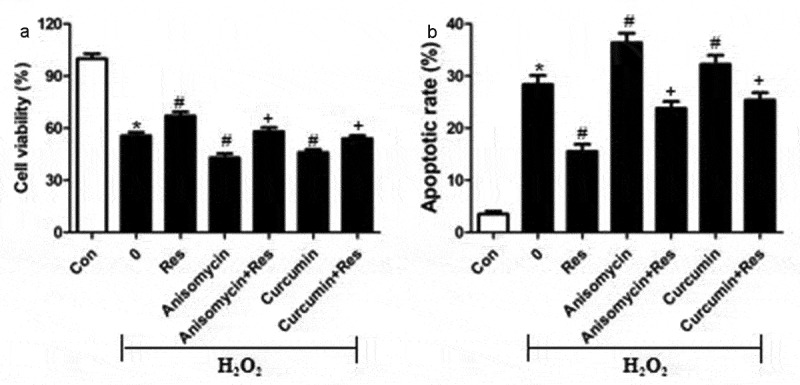
MAPK signaling agonists exacerbate the pro-apoptotic effects of H_2_O_2_ on RGC-5 cells. cells were preincubated for 4 h with resveratrol (Res, 10 μM), anisomycin (1.5 μM) or curcumin (1.5 μM), or the combination of resveratrol with each agonist. Then cells were treated for 24 h with 200 μM H_2_O_2_. (a) Cell viability was detected using the CCK8 assay (b) apoptosis was analyzed using flow cytometry. *P< 0.05 vs control group, ^#^*P*< 0.05 vs H_2_O_2_-treated group, ^+^*P*< 0.05 vs H_2_O_2_+ Res group

To test the hypothesis that resveratrol inhibits H_2_O_2_-induced RGC-5 cell death by suppressing MAPK signaling, we exposed cells to H_2_O_2_ in the presence of the p-38 inhibitor PD169316, ERK inhibitor FR180204 or JNK inhibitor SP600125, then examined cell viability and apoptosis. Certain cultures were treated with each of these inhibitors in the presence of resveratrol as well. Not only did each of these inhibitors substantially reverse H_2_O_2_-induced cell apoptosis and death, but they enhanced the effects of resveratrol (Figure 6a and b). Conversely, we found that either anisomycin, an agonist of JNK and p38, or the ERK agonist curcumin exacerbated the pro-death and pro-apoptosis effects of H_2_O_2_, and that resveratrol reversed these effects (Figure 7a and b). These results are consistent with the idea that resveratrol protects retinal ganglion cells against H_2_O_2_-induced apoptosis by suppressing MAPK cascades.

## Discussion

Here we provide *in vitro* evidence that resveratrol can reduce the oxidative stress, apoptosis and cell death of RGG cells induced by exposure to H_2_O_2_. We further show that these protective effects may involve inhibition of MAPK signaling. These findings help clarify how glaucoma may progress and identify MAPK pathways as potential therapeutic targets to mitigate or even prevent the disease, and the results demonstrate the drug potential of resveratrol.

Death of RGG cells is closely related to glaucoma, and ROS production is a major driver of apoptosis of these cells [16,17]. Consistent with these findings, we found that exposing RGC-5 retinal ganglion cells to 200 μM H_2_O_2_ suppressed the activities of SOD, CAT, and GSH and decreased cell viability. The imbalance of pro- and anti-oxidant system could lead to oxidative stress. In clinical study, the levels of ROS were found to be significantly increased in patients with glaucoma [18]. We found that resveratrol increased the activities of SOD, CAT, and GSH, while it decreased ROS production in H_2_O_2_-induced RGC-5 cells. In addition, it reversed H_2_O_2_-induced loss of Δψm, thereby reversing a sign of ROS-mediated mitochondrial dysfunction [19] and early apoptosis [20]. Indeed, we found that H_2_O_2_ increased levels of cleaved caspases-3 and −9, the main initiators and drivers of apoptosis [21], while resveratrol reversed this. These findings indicate that resveratrol possesses potent anti-oxidant activities against H_2_O_2_-induced damage in retinal ganglion cells.

The activation of MAPKs signal transduction contributes to cell differentiation, growth, and immune regulation [22]. The members of MAPKs include ERK, JNK, and p38 [23]. MAPKs cascades were found to be involved in regulating intracellular metabolism and the adaptive responses to stresses which leads to oxidative injury [24]. Our study begins to elucidate the mechanism of therapeutic action of resveratrol against glaucoma by showing that it inhibits the activation of ERK, JNK, and p38 signaling cascades in H_2_O_2_-induced RGC-5 cells. These three MAPKs regulate intracellular metabolism and respond to external stress [25,26]. We confirmed our results using MAPK inhibitors and agonists. Our results extend the literature showing that resveratrol inhibits the MAPK signaling pathway in other cell lines, thereby alleviating oxidative stress [27–29]. Similar to our results, resveratrol has an ability to therapy tumor via suppressing JNK, ERK, and p38 signaling pathways [30–32]. In addition, previous study also reported that LAMA4 down-regulation may decrease the ROS-stimulated death of RGG cells via suppressing the activation of the MAPKs cascade [33]. A p38 MAPK inhibitor has been also reported to improve outcome after glaucoma filtration surgery in clinic or in animals [34,35], suggesting that the activation of MAPKs cascade contributes to the occurrence and development of glaucoma. Thus, MAPKs could be as an ideal candidate for the treatment of glaucoma.

In this way, our work demonstrates that resveratrol can protect RGG cells against oxidative stress-stimulated apoptosis by inhibiting MAPK pathways. Our data establish the therapeutic potential of this natural product against glaucoma. Furthermore, the detailed mechanisms in interaction between resveratrol and MAPK pathways should be further explored.

## Conclusion

In summary, resveratrol has the potential to decrease the oxidative stress injury in retinal ganglion cells through inhibiting MAPK signaling cascades. Thus, it may be a treatment option for preventing glaucoma.

## HIGHLIGHTS

1. Resveratrol inhibits the oxidative stress damage in retinal ganglion cells.

2. Resveratrol suppresses the activation of MAPKs signaling pathways.

3. Resveratrol shows potential for preventing glaucoma.
